# LPS- and LTA-Induced Expression of IL-6 and TNF-***α*** in Neonatal and Adult Blood: Role of MAPKs and NF-***κ***B

**DOI:** 10.1155/2014/283126

**Published:** 2014-10-30

**Authors:** Lutz Koch, David Frommhold, Kirsten Buschmann, Navina Kuss, Johannes Poeschl, Peter Ruef

**Affiliations:** ^1^Department of Neonatology, University Children's Hospital, Medical School, University of Heidelberg, 69120 Heidelberg, Germany; ^2^Department of Neonatology, Catholic Children's Hospital Wilhelmstift, 22149 Hamburg, Germany; ^3^Clinic of Pediatrics, SLK-Kliniken Heilbronn, 74078 Heilbronn, Germany

## Abstract

As nuclear factor kappa B (NF-*κ*B) and mitogen-activated protein kinases (MAPKs) seem to be critical mediators in the inflammatory response, we studied the effects of lipopolysaccharide (LPS) and lipoteichoic acid (LTA) on (a) the activation of NF-*κ*B and MAPKs and (b) the expression of tumor necrosis factor alpha (TNF-*α*) and interleukin 6 (IL-6) with or without the specific inhibitors of these intracellular signal transduction pathways in neonatal cord and adult blood. TNF-*α* and IL-6 concentrations showed a sharp increase in the supernatants of cord and adult whole blood after stimulation. TNF-*α* concentrations were significantly higher, whereas IL-6 concentrations were tendentially lower in adult blood after stimulation. Stimulation with LPS or LTA resulted in a significantly decreased activation of p38 MAPK in neonatal compared with adult blood. Although LTA failed to induce additional ERK1/2 phosphorylation, LPS stimulation mediated the moderately increased levels of activated ERK1/2 in neonatal monocytes. The addition of the p38 MAPK inhibitor SB202190 significantly decreased IL-6 and TNF-*α* production upon LPS or LTA stimulation. Furthermore, the inhibition of ERK1/2 was able to reduce LPS-stimulated TNF-*α* production in neonatal blood. We conclude that p38 MAPK as well as ERK1/2 phosphorylation is crucially involved in LPS activation and could explain the differences in early cytokine response between neonatal and adult blood.

## 1. Introduction

Sepsis and its associated syndromes of systemic inflammatory response and multiple organ dysfunction continue to be leading causes of morbidity and mortality in newborns [1]. Most clinical signs observed in the initial phase of sepsis are thought to be triggered by the activation of a Toll-like receptor (TLR) [2]. The interaction between TLRs and microbial antigens (components) initiates the activation of an evolutionary conserved immune signaling network, leading to the rapid and transient phosphorylation of several downstream signaling proteins. Lipopolysaccharide (LPS) from Gram-negative bacteria and lipoteichoic acid (LTA) from Gram-positive bacteria are major immunostimulatory bacterial cell wall components and activate the mitogen-activated protein kinases (MAPKs) p38, the extracellular-regulated kinase 1/2 (ERK1/2), and the Jun N-terminal kinase (JNK) in a myeloid differentiation factor 88- (MyD88-) dependent pathway through TLR4 and TLR2, respectively [3–7]. The ultimate outcome is the transcription of hundreds of inflammatory mediators. p38 MAPK crucially mediates the release of proinflammatory cytokines by the regulation of the expression of a variety of genes, which are involved in the acute-phase response [8–10]. p38 MAPK inhibition shows broad anti-inflammatory effects in human endotoxemia, and delayed administration can improve lethality from cecal ligation and puncture [11–13].

As MAPKs, nuclear factor kappa B (NF-*κ*B) acts as a critical step through the regulation of genes encoding proinflammatory cytokines such as tumor necrosis factor alpha (TNF-*α*), chemokines, adhesion molecules, and other inducible enzymes [14]. The inappropriate and prolonged activation of NF-*κ*B has been linked to several diseases associated with inflammatory events, including septic shock, acute respiratory distress syndrome, ischemia, and reperfusion injury [15].

We have previously shown that the inhibition of LPS-induced p38 MAPK activation in neonatal and adult whole blood was associated with a strong reduction in the release of cytokines, whereas the pharmacological inhibition of NF-*κ*B showed no effect. Furthermore, stimulation with LPS resulted in a significantly decreased activation of p38 MAPK in neonatal blood [16].

We assume that the decreased functional response of the newborn immune system to LPS and LTA may be due to differences in its signaling pathways compared with those of adults. NF-*κ*B and MAPKs seem to be critical mediators in the inflammatory response. Therefore, we studied the effects of LPS and LTA on the expression of NF-*κ*B and MAPKs in neonatal cord and adult blood monocytes. Furthermore, we investigated the impact of the specific inhibitors of these intracellular signal transduction pathways on interleukin 6 (IL-6) and TNF-*α* expression after LPS and LTA stimulation.

## 2. Materials and Methods

### 2.1. Blood Sampling

Peripheral blood was obtained from 10 healthy adult volunteers. Cord blood samples were obtained by puncturing with sterile needles the umbilical cords of seven healthy, full-term infants after a scheduled cesarean section. Newborns did not show any signs of bacterial infection during a follow-up of 1 week.

After discarding the first 2 mL, blood was collected in lithium-heparin tubes (S-Monovette; Sarstedt, Nümbrecht, Germany), and samples were immediately used for experiments. Blood sampling was performed in accordance with the principles of the Declaration of Helsinki. The study was approved by the local ethics committee.

### 2.2. Incubation of Blood with LPS and LTA

In all experimental series, the incubation of blood was performed at 37°C. All samples were rested for 30 min. In flow-cytometry experiments, whole neonatal cord and adult blood were incubated with different concentrations of LPS (0.1, 1, 10, 100, and 1000 ng/mL), LTA (0.1, 1, 10, 100, and 1000 *μ*g/mL), or vehicle for 15 min. In enzyme-linked immunosorbent assay (ELISA) experiments, whole neonatal cord and adult blood samples were incubated with 20 *μ*M of the NF-*κ*B inhibitor BAY11-7082 (Merck, Hull, UK), 20 *μ*M of the p38 MAPK inhibitor SB202190 (Merck), 20 *μ*M of the ERK1/2 inhibitor U0126 (Enzo Life Sciences, Lörrach, Germany), or 20 *μ*M of the JNK inhibitor SP600125 (Merck) for 30 min. Thereafter, 100 ng/mL LPS or 100 *μ*g/mL LTA was added to the pretreated samples, and the whole blood was incubated for 4 h.

### 2.3. Flow-Cytometric Analysis of NF-*κ*B, p38, ERK1/2, and JNK

The phosphorylation of NF-*κ*B and p38 MAPK in newborn and adult blood in response to LPS or LTA was determined according to a modified eBioscience protocol for staining intracellular antigens (Protocol B). In brief, 200 *μ*L whole blood samples were incubated with 6 *μ*L fluorescein isothiocyanate- (FITC-) conjugated mouse anti-CD14 antibody at 37°C for 30 min. After exposure to different stimuli (15 min), the experiments were stopped by the addition of 1.9 mL Fix/Lyse buffer (BD Biosciences, San Jose, CA, USA) according to the manufacturer's protocol. After one wash, cells were permeabilized by adding 500 *μ*L Foxp3 Fixation/Permeabilization buffer (eBioscience, San Diego, CA, USA) and incubated for 45 min on ice.

After washing with 2 mL of 1× permeabilization buffer, cells were stained with 20 *μ*L Alexa Fluor 647-labeled anti-p38 MAPK antibody (BD Biosciences), 22 *μ*L PE-labeled anti-NF-*κ*B p65 (pS529) antibody (BD Biosciences), 6 *μ*L PE-labeled anti-ERK1/2 (pT202/pY204) (BD Biosciences), and/or Alexa Fluor 647-labeled mouse anti-JNK (Thr183/Tyr185) (New England Biolabs, Ipswich, MA, USA) for 30 min at room temperature. Finally, after washing with permeabilization buffer, cells were measured by flow cytometry with an LSR II (BD Biosciences), and data were analyzed using FlowJo 10.0.5 software (TreeStar Inc., Ashland, OR, USA).

### 2.4. Enzyme-Linked Immunosorbent Assay

Supernatants were collected, and the production of IL-6 and TNF-*α* was measured by sandwich ELISA assay obtained from R&D Systems (Minneapolis, MN, USA). ELISA was performed according to the manufacturer's protocol.

### 2.5. Statistical Analysis

All results are presented as mean and standard deviation. The normality distribution was tested using the Kolmogorov-Smirnov test, showing that all variables were normally distributed. The results were evaluated using Student's *t*-test or one-way analysis of variance. Statistical significance was set at *P* < 0.05. When the difference was significant, multiple-comparison post hoc tests were performed to determine which group or groups were different. Using Levene's test of homogeneity of variance, we selected the appropriate post hoc test. For homogeneous group variances, the Tukey HSD test was used, or a Tamhane T2 test was performed. All analyses were done using SPSS version 16.0 (SPSS Inc., Chicago, IL, USA).

## 3. Results

### 3.1. Levels of IL-6 and TNF-*α* Cytokine Production in the Supernatants of Whole Neonatal Cord and Adult Blood after LPS and LTA Stimulation

**Figure 1:**
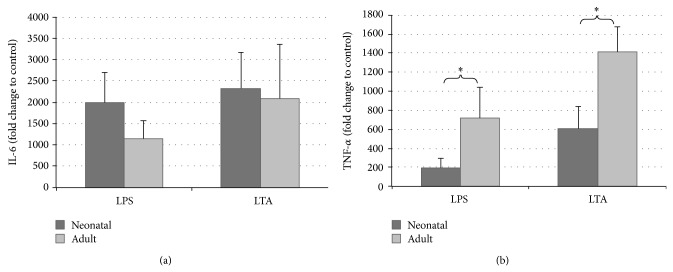
Comparison of the in vitro stimulation of neonatal and adult whole blood (WB) with lipopolysaccharide (LPS) and lipoteichoic acid (LTA). Human WB was stimulated in parallel with LPS (100 ng/mL) and LTA (100 *μ*g/mL) or left unstimulated for 4 h. Supernatants were then collected, and IL-6 (a) and TNF-*α* (b) were analyzed using ELISA. Data are presented as fold difference as compared to untreated controls. Values are expressed as mean ± standard error of mean (SEM). ^*^ *P* < 0.05, neonatal versus adult.

Figure 1 shows the expression change of TNF-*α* and IL-6 after LPS and LTA stimulation. TNF-*α* and IL-6 concentrations showed a sharp increase in the supernatants of cord and adult whole blood after LPS or LTA stimulation for 4 h. TNF-*α* concentrations were significantly higher, whereas IL-6 concentrations were tendentially lower in adult blood after LPS or LTA stimulation.

### 3.2. Effects of the Specific Inhibitors of NF-*κ*B, p38, ERK1/2, and JNK on IL-6 and TNF-*α* Cytokine Production in the Supernatants of Whole Neonatal Cord and Adult Blood after LPS and LTA Stimulation

**Figure 2:**
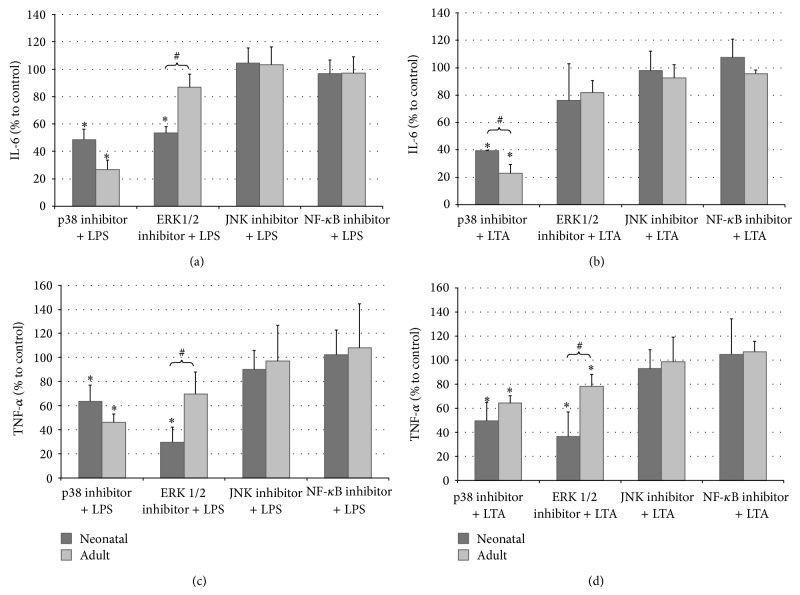
In vitro lipopolysaccharide (LPS) and lipoteichoic acid (LTA) stimulation of neonatal and adult whole blood (WB) in the presence of specific inhibitors. Human WB was preincubated with 20 *μ*M of the p38 MAPK inhibitor SB202190, 20 *μ*M of the ERK1/2 inhibitor U0126, 20 *μ*M of the JNK inhibitor SP600125, or 20 *μ*M of the NF-*κ*B inhibitor BAY11-7082, followed by LPS (100 ng/mL) ((a) and (c)) or LTA (100 *μ*g/mL) ((b) and (d)) for an additional 4 h. Supernatants were then collected, and IL-6 ((a) and (b)) and TNF-*α* ((c) and (d)) were analyzed using ELISA. Data are presented as fold difference as compared to untreated controls. Values are expressed as mean ± standard error of mean (SEM). ^*^ *P* < 0.05, control versus inhibitor. ^#^ *P* < 0.05, neonatal versus adult.

The role of MAPK p38 in the observed cytokine production in LPS- or LTA-stimulated whole neonatal and adult blood was examined by incubation with the p38 kinase inhibitor SB202190. Coincubation with SB202190 significantly decreased the LPS- or LTA-induced IL-6 and TNF-*α* production in adult as well as neonatal whole blood (Figure 2). However, the inhibition effect of SB202190 on IL-6 expression was substantially higher in adults than that in newborns, whereas the effect on TNF-*α* expression resulted in similar values for newborn and adults.

In order to determine the role of the MAPK ERK1/2 in the response of neonatal and adult blood after LPS or LTA stimulation, we added the MAPK ERK1/2 inhibitor U0126. U0126 was shown to inhibit IL-6 and TNF-*α* secretion only in neonatal blood after LPS stimulation. Interestingly, U0126 did not affect cytokine secretion in adult blood and after LTA stimulation (Figure 2).

When whole neonatal and adult blood were preincubated with BAY11-7082, a specific inhibitor of NF-*κ*B, or SP600125, a specific inhibitor of JNK, and stimulated with LPS or LTA, no significant difference was observed when compared with controls (Figure 2).

### 3.3. Effects of LPS and LTA on NF-*κ*B and MAPKs p38, ERK1/2, and JNK Phosphorylation in Neonatal Cord and Adult Monocytes

**Figure 3:**
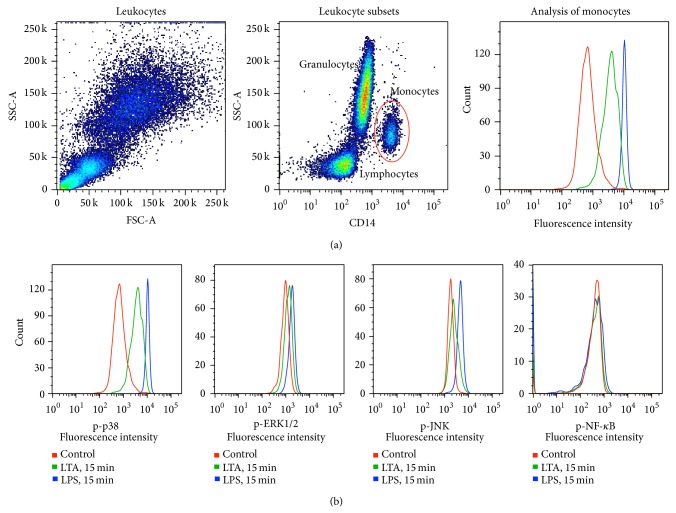
Flow-cytometric analysis of p38, ERK1/2, JNK, and NF-*κ*B in monocytes after lipopolysaccharide (LPS) and lipoteichoic acid (LTA) stimulation in vitro. (a) After the stimulation of whole blood (WB) in vitro, the red blood cells were lysed, and the leukocytes were fixed and permeabilized. The leukocytes were stained with modification-specific fluorochrome-conjugated antibodies and an antibody against the CD14 monocyte cell surface marker. The leukocyte population was separated into lymphocytes, granulocytes, and monocytes based on differences in side scatter (granularity) and binding to the CD14 antibody using flow cytometry. The CD14-positive monocyte population was analyzed for the intracellular levels of phosphorylated signaling proteins based on the determination of mean fluorescence intensity (MFI). (b) Measurements of fluorescence intensity from the monocyte subset after staining with the fluorochrome-conjugated antibodies against phosphorylated p38, ERK1/2, JNK, and NF-*κ*B displayed as histograms. The figure shows a fluorescence intensity of unstimulated controls and samples stimulated with LPS and LTA for 15 min.

The flow-cytometric analysis after staining of cell surface markers in combination with antibodies against intracellular protein enables the analysis of signaling events at the single-cell level in complex cell populations [17]. The effects of LPS and LTA on the phosphorylation level of MAPKs and NF-*κ*B were evaluated in the neonatal and adult monocyte subset after the stimulation of whole blood for 15 min in vitro. On the basis of differences in light-scattering properties and binding of an antibody against the monocyte cell surface marker cluster of differentiation 14 (CD14), the complex leukocyte population was separated into granulocytes, lymphocytes, and monocytes after the stimulation of whole blood. The CD14-positive monocyte population was analyzed for the intracellular levels of phosphorylated signaling proteins based on the determination of mean fluorescence intensity (MFI) (Figure 3).

**Figure 4:**
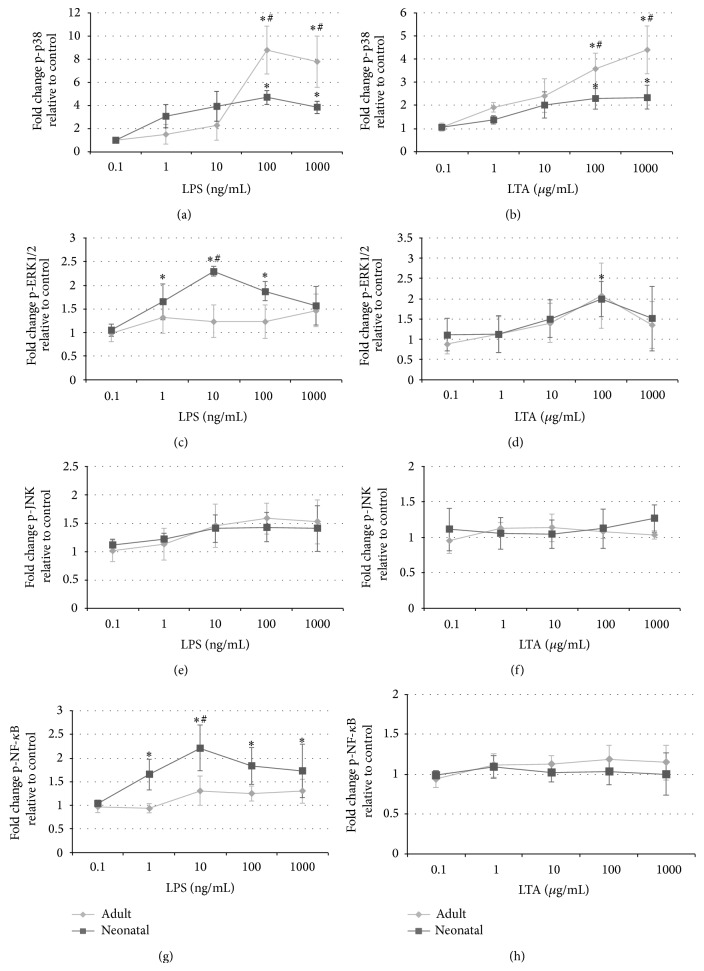
Effects on phosphorylated p38 MAPK, ERK1/2, JNK, and NF-*κ*B in monocytes after stimulation with lipopolysaccharide (LPS) and lipoteichoic acid (LTA) of whole blood (WB) in vitro. WB was stimulated with LPS (100 ng/mL) or LTA (100 *μ*g/mL) for 15 min. The red blood cells were lysed, and the leukocytes were fixed and permeabilized. The samples were stained with antibodies against CD14 (FITC-conjugated), phosphorylated p38 MAPK (pT180/pY182; Alexa Fluor 647 conjugated), ERK1/2 (pT202/Y204; PE-conjugated), JNK (Thr183/Tyr185; Alexa Fluor 647 conjugated), and/or NF-*κ*B p65 (pS529; PE labeled) and analyzed for the intracellular levels of phosphorylated signaling proteins based on the determination of MFI by flow cytometry. Stimulated samples were compared with unstimulated controls, and the results were expressed as fold change in phosphorylation: ^*^ *P* < 0.05, LPS or LTA versus control. ^#^ *P* < 0.05, neonatal versus adult.

The stimulation of blood with LPS and LTA dose-dependently induced the phosphorylation of p38 MAPK within 15 min in both newborn and adult monocytes (Figures 4(a) and 4(b)). The effect was more pronounced after LPS than LTA stimulation. However, the peak level of p38 MAPK phosphorylation was substantially higher in adults than that in newborns.

Furthermore, the analysis of the monocyte subset showed that LTA failed to induce additional ERK1/2 phosphorylation, whereas LPS stimulation mediated the increased levels of activated ERK1/2 in neonatal but not in adult monocytes (Figures 4(c) and 4(d)).

None of the stimuli were demonstrated to mediate changes in p-JNK or p-NF-*κ*B levels (Figures 4(e)–4(h)).

## 4. Discussion

Although it has been assumed that neonatal cytokine responses are generally impaired, recent evidence suggests that the pattern of stimulus-induced neonatal cytokine production is cytokine specific, revealing a Th2 bias of the neonatal immune response. Little is known about the molecular basis for these differences in cytokine production between newborns and adults. NF-*κ*B and MAPKs have been implicated as critical mediators of the release of inflammatory cytokines and regulate the expression of a variety of genes involved in the acute-phase response such as TNF-*α*, IL-6, and other inducible enzymes [8, 9, 18].

We have previously shown that stimulation of whole blood with LPS (100 ng/mL) induced a peak phosphorylation of p38 MAPK and NF-*κ*B within 15 min in both newborn and adult cells, with a significantly decreased activation of p38 MAPK in neonatal blood [16, 19]. The inhibition of LPS-induced p38 MAPK activation in neonatal and adult whole blood was associated with a strong reduction in the release of cytokines, whereas the pharmacological inhibition of NF-*κ*B showed no effect.

In the present study, we present more data that emphasize and extend these earlier observations. We compared monocytes from cord blood with monocytes from peripheral blood of adults in terms of their functional response to the ligands of TLR4 and TLR2 (i.e., LPS and LTA) on the level of signal transduction (MAPKs and NF-*κ*B) after 15 min and cytokine secretion (IL-6 and TNF-*α*) after 4 h, to reflect the early activation of TLR-mediated signal transduction pathway.

We focused our investigation on monocytes because TLR2 and TLR4 seem to be mainly expressed on monocytes rather than granulocytes. Accordingly, we found a very modest activation of MAPKs in whole blood granulocytes on challenge with LPS or LTA compared to that in monocytes (data not shown). The responses to bacterial products were determined using a whole blood approach. This approach keeps the blood immune cells in their natural environment, and all factors and cofactors involved in innate immune response are present. Furthermore, no artifacts due to isolation procedures are to be expected, and data should most likely mirror the in vivo situation [20].

Interestingly, we showed that the induction of TNF-*α* by LPS as well as LTA was strikingly reduced in neonatal cord blood, whereas IL-6 concentrations were tendentially higher. Angelone et al. [21] demonstrated a strong Th2 bias of TLR-mediated neonatal monocyte cytokine responses in vitro and a low TNF-*α* production and high IL-6/TNF-*α* ratios during the first days of life among healthy and infection-exposed newborns in vivo. After stimulation with heat-killed* Escherichia coli* or group B *β*-hemolytic streptococci, Mohamed et al. [22] detected significantly higher concentrations of IL-6 in neonatal blood than in the samples from adults.

Polarization in favor of cytokines with anti-inflammatory and Th2 polarization properties may protect the fetus in utero from potentially harmful Th1 responses that can trigger spontaneous abortion [23] as well as premature delivery and its consequences [24]. In addition, IL-6 is an important inducer of the acute-phase response and may thereby play an important protective role. However, given the importance of Th1-polarizing cytokines in protection against multiple pathogens, impairment in perinatal TNF-*α* production may render the newborns more susceptible to microbial infection.

Simultaneously, our flow-cytometric analysis and series of inhibitor tests showed the importance of the MAPK p38 for TNF-*α* secretion after LPS or LTA stimulation. The peak level of phosphorylation was substantially lower in neonates than that in adults, and selective p38 inhibition significantly decreased the levels of TNF-*α* and IL-6. Several authors have implicated the MAPK p38 to be important for monocyte function. Meja et al. [25] have determined that LPS-generated granulocyte-macrophage colony-stimulating factor and TNF-*α* secretion depended on a functional MAPK p38 [26].

Rutault et al. [27] showed that both p38 and ERK pathways are necessary for normal LPS-induced TNF-*α* mRNA accumulation in cells. We demonstrated that the inhibition of ERK1/2 was able to ablate LPS-stimulated TNF-*α* induction in neonatal blood. Furthermore, LPS stimulation mediated the increased levels of activated ERK1/2 in neonatal monocytes only. In contrast to p38 MAPK, which is important for early TNF-*α* production in adult as well as neonatal monocytes, it seems that ERK1/2 is relevant in neonatal monocytes.

We resolve that phosphorylation of both p38 MAPK and ERK1/2 is a crucial step in the early LPS- or LTA-triggered activation of blood cells, which tends to be more important in neonates than in adults. This, in turn, could explain the differences in the early cytokine response between neonatal and adult blood. The role of ERK1/2 is not fully understood until now. Furthermore, we assume that NF-*κ*B activation and MAPK JNK play a secondary role in LPS-mediated early cytokine production.

## 5. Conclusion

We found a markedly reduced TNF-*α* response to LPS or LTA in neonatal compared with adult blood, whereby p38 MAPK and ERK1/2 may be important regulators. Because inflammation is an important trigger of premature delivery and its attendant complications [28], we speculate that there may be a selective advantage to having impaired neonatal p38 MAPK activation in utero to reduce the risk of a deleterious immune response toward maternal and microbial molecules. Such p38 MAPK-mediated suppression of TLR4 and TLR2 signaling may protect against an overwhelming immune reaction but may also lead to a higher risk of infection for the fetus and newborn.
